# Assessing recall bias and measurement error in high-frequency social data collection for human-environment research

**DOI:** 10.1007/s11111-019-0314-1

**Published:** 2019-02-07

**Authors:** Andrew Bell, Patrick Ward, Md. Ehsanul Haque Tamal, Mary Killilea

**Affiliations:** 1 Department of Environmental Studies, New York University, 285 Mercer St., New York, NY 10003, USA; 2 Master of Environmental Policy Program, Duke Kunshan University, No. 8 Duke Avenue, Kunshan, Jiangsu 215316, China; 3 International Food Policy Research Institute (IFPRI), 1201 Eye St, NW, Washington, DC 20005-3915, USA; 4 icddr,b, 68, Shaheed Tajuddin Ahmed Sarani, Mohakhali, Dhaka 1212, Bangladesh; 5 International Food Policy Research Institute (IFPRI), Dhaka Office, House 10A, Road 35, Gulshan 2, Dhaka 1212, Bangladesh

**Keywords:** High-frequency data collection, Android smartphone, Microtasks for micropayments, Bangladesh

## Abstract

A major impediment to understanding human-environment interactions is that data on social systems are not collected in a way that is easily comparable to natural systems data. While many environmental variables are collected with high frequency, gridded in time and space, social data is typically conducted irregularly, in waves that are far apart in time. These efforts typically engage respondents for hours at a time, and suffer from decay in participants’ ability to recall their experiences over long periods of time. Systematic use of mobile and smartphones has the potential to transcend these challenges, with a critical first step being an evaluation of where survey respondents experience the greatest recall decay. We present results from, to our knowledge, the first systematic evaluation of recall bias in components of a household survey, using the Open Data Kit (ODK) platform on Android smartphones. We tasked approximately 500 farmers in rural Bangladesh with responding regularly to components of a large household survey, randomizing the frequency of each task to be received weekly, monthly, or seasonally. We find respondents’ recall of consumption and experience (such as sick days) to suffer much more greatly than their recall of the use of their households’ time for labor and farm activities. Further, we demonstrate a feasible and cost-effective means of engaging respondents in rural areas to create and maintain a true socio-economic “baseline” to mirror similar efforts in the natural sciences.

One of the most significant challenges impeding our understanding of human-environment interactions is that quantitative data on social systems are not collected in a manner comparable to our collection of natural systems data. This is particularly true in light of recent advancements in researchers’ ability to collect data on natural systems through digital sensors. Sensor technologies—from very site-specific moisture and flow sensors up to very remote satellite-based sensors—allow for measurement of the natural environment that is both temporal in scope, and, where applicable and modeling approaches allow, spatially explicit (e.g., Elliott et al. 2015; Haerter et al. 2015; Döll and Fiedler 2007; Harris et al. 2014). The spatio-temporal nature of these new data streams allow scientists and decision-makers to examine environmental disturbances in terms of “anomalies”—deviations of some variable like sea surface temperature (e.g., Collins et al. 2013; Döll 2009) or soil moisture (Funk et al. 2015; Enenkel et al. 2016) from the long-term average that can be derived from this regular data baseline. To date, the same lens into environmental disturbance has not been possible with social systems data. While social data collection is often at regular time intervals (e.g., annual) and (at best) geographically representative, the expense and logistic challenge of these efforts precludes data collection at the frequency necessary to capture responses to environmental disturbance by human systems.^1^ Consider, for example, the emblematic human-environment problem of food security. In discussing some of the measurement challenges associated with food security assessments, Headey and Ecker (2013) note that “First, decision-makers need to make a wide range of cross-sectional ‘snapshot’ comparisons: between different social groups, different regions, and different countries. Second, decision-makers need different sorts of inter-temporal comparisons: on long-term trends, on the seasonality of food insecurity, and on the impacts of shocks, such as droughts, floods, or changes in incomes and prices.” High-frequency data collection of human-environment response does occur, but it is typically a “fire alarm” (McCubbins and Schwartz 1984) response to crises already in progress, thereby precluding comparisons to normal or baseline conditions and the factors that prevented droughts, floods, or other hazards from becoming crises in other places and times.

The nascent practice of data collection from mobile devices—in systematic survey research, distinct from citizen science (e.g., Overeem et al. 2013) and market research (e.g., Jana 2018)—provides an opportunity to correct this gap and allow for high-velocity (Shekhar et al. 2017) socio-economic baselines, largely by reducing the cost barriers of social data collection. Resources typically used for fuel, logistics, lodging, and wages for trained enumerators can be channeled directly to respondents as mobile talk time, data, device ownership, or other incentive (Bell et al. 2016). Regular engagement via short tasks (e.g., survey items, economic games, or various forms of micro-experiments) can provide a high-frequency, representative, socio-economic baseline in a cost-effective manner. All this does not come free of charge, so it is important to develop an understanding of what streams of data are worth knowing with high frequency, and what gains are possible relative to a conventional, much more infrequent survey approach, taking into consideration data losses due to missed intra-period variation and difficulty in recall. To the best of our knowledge, the present study represents the first use of high-frequency data collection to systematically compare recall bias across a range of typical survey tasks. Among other novel features of our data, we randomized how frequently participants were asked a particular survey task, which ultimately provided task-specific estimates of changes in recall bias (as shift in average, and loss of variation) as task frequency is increased from once per season, to once per month, to once per week.

## The many dimensions of recall bias

Data collection from rural areas, particularly in developing countries, embodies the challenges in generating meaningful socio-economic baselines. Typical surveys “are conducted at one point in time, often with lengthy recall periods,” (Carletto et al. 2010), potentially missing or misestimating important decisions that are made continually along a season, such as investment in irrigation, application of pesticides, or the time spent weeding. Further, these “one point in time” surveys typically take weeks or months to undertake, so that, in practice, respondents face different challenges in recalling activities from a particular point in time (e.g., before the June harvest). This particular challenge may not generally bias results. For example, McCullough (2017) found no evidence of the timing of survey interviews biasing assessments of annual labor productivity in either Tanzania or Uganda and only minor effects in Malawi. However, problems of recall can be compounded with problems of sampling when respondents report on a fixed, relative recall period (e.g., food consumption over the past 30 days; Qaim and Kouser 2013), resulting in respondents in practice reporting on different intervals of time, and potentially under very different conditions.^2^

Cognitively, reporting of past activities is potentially subject to a range of different recall errors: telescoping (where respondents incorrectly shift activity forward or backward in time, into or out of the recall period), heaping (where respondents incorrectly agglomerate past events into one point in time, e.g., “about 3 months ago”), and recall decay (where events further in the past are forgotten and under-reported) errors (Beegle et al. 2012a). These recall errors are perhaps so pervasive and systematic as to give rise to substantive recall biases, as opposed to simple random anomalies that might be smoothed out over respondents. Further, they can be compounded in panel (repeated visit) surveys in what are called “seam effects” (Tourangeau et al. 2010), where apparent differences between two adjacent time periods reported on in different survey waves emerge due only to differences in recall. Further dimensions of bias can be imposed by the structure of the questions themselves. As an example, wages in the World Bank’s Living Standards Measurement Surveys-Integrated Surveys of Agriculture (LSMS-ISA) are collected as a 12-month recall with respondents asked for the typical number of weeks per month worked and typical hours per week (McCullough 2017). In this framing, “[s]ystematic measurement error in construction of labor supply variables is particularly concerning, should respondents recall different types of activities with different errors” (McCullough 2017). Hiroyuki and Mari (2009) observed the same problem among Vietnamese respondents to a livelihoods survey, who exhibited significant bias in reporting expenditure in specific categories; bias was much lower in the reporting of aggregate expenditure. Over longer recall periods, some tasks are more challenging than others to recall, while other tasks may be easier. In part, this can be shaped by related factors that can make particular events more salient than others. For example, in some parts of Sub-Saharan Africa, hiring non-family labor to work on the farm is rare, and the burden of making payment for labor helps the event to “stand out” in survey respondents’ memory (Beegle et al. 2012a).

Finally, the structure of the survey can shape bias in recall. Meyer et al. (2009), for example, find under-reporting of government transfers across a range of large-scale surveys (even including large, well-known surveys in the USA like the Survey of Income and Program Participation, SIPP, and the Panel Study of Income Dynamics, PSID), and suggest that even the desire to shorten what can be very long interviews may be systematically biasing results (Meyer et al. 2009).

## Toward rural diaries

The obvious solution to recall problems—conducting short, regular, “diary” style surveys with very short recall periods for sums of consumption, income, and labor efforts—happens commonly in developed country settings, but can be logistically prohibitive in rural settings in developing economies (Hiroyuki and Mari 2009).^3^ Where short-recall approaches have been evaluated in these contexts, results have been promising. Comparing a range of different recall periods, Beegle et al. (2012b) measured 7-day recall of consumption and deemed the resulting data to be better matched with benchmark data (regular, event-driven diaries of consumption) than those based on 14-day recall, though still subject to telescoping errors and recall bias. Information and communication technologies (ICTs) such as mobile and smartphones have the potential to make diaries (or at least, short-recall surveys) feasible in these rural contexts, as the number of mobile and broadband subscriptions soars (ITU 2016). An obvious benefit of these technologies in this setting is the ability to deploy a large number of surveys for very little cost (our own experiment, for example, elicited survey task responses for as little as USD 0.1 per data point; Bell et al. 2016, and “Experimental design” in this current study). In addition, modern accessibility to mobile devices implies that in a very near future, mobile surveys could potentially be more representative than traditional surveys, as respondents are able to respond in their own time without needing to take half or whole day away from work in order to have their voices heard (Grossman et al. 2014). While Headey and Ecker (2013) remark that the “expensive solution to [the food security monitoring] problem would be to conduct household surveys in higher frequency[,]” they also opine that ICTs should bring down the expense of engagement with rural communities significantly, bringing benefits that far outweigh costs. In the present study, this is precisely what we aim to demonstrate.

## Methods

We conducted a 50-week longitudinal study of 480 farmers^4^ in Rangpur District in northern Bangladesh via Android smartphones. Farmers responded to short survey tasks (designed to consume nor more than approximately 3-5 min each) administered in the Open Data Kit (ODK) platform (Brunette et al. 2013), launched via a custom, user-friendly app, on a regular basis (approximately 5-10 tasks per week). In exchange for completing these survey tasks, participants received small payments of mobile talk time, data, and credit toward ownership of the handset (Symphony Roar V25, with market value of approximately $45 at the time of the survey). In the next sections, we explain our sampling approach, outline the detailed design of this experiment, and finally describe the specific analysis applied in the current study.

### Sampling frame and strategy

Within rural Rangpur, our goal was to draw a sample of plausible—or even likely— early adopters of smartphone technology. The rationale for this is simple: while this technology has great potential, the specific approach we were piloting was unproven, and thus susceptible to inadvertent sabotage from, among other potential sources, a sample of farmers unprepared to fully engage with the pilot (e.g., due to illiteracy or unfamiliarity with mobile technologies). Our sampling frame consisted of a pooled list of villages from the two (out of a total of eight) upazilas (subdistricts) of Rangpur with the highest literacy rates reported in the most recently available census (2011). We randomly selected 40 villages total, and for each village, we requested a short list of 25 potential participants from the agricultural extension officer responsible for the village, based on their assessment of technical capacity (i.e., observed use of mobile phones and inclination to use a smartphone). From each list, we randomly selected 12-13 participants, with the goal of minimizing any patronage given in adding individuals to the list. This procedure ultimately yielded a final sample of 480 participants across the 40 randomly selected villages. On average, our sample was younger, more literate, and more educated than the average person from Rangpur Division, and skewed much further toward male respondents (Table 1).

**Table 1:** Sample characteristics, compared against a representative Rangpur Sample, and the Bangladesh average

	(1)	(2)	(3)
Demographic variable	This study (2016)	Rangpur division (household-head) (2011) (Ahmed 2013)	Bangladesh average (2015)
Average age	32.9 (11.8)	44.2 (13.79)	26.3*^1^
Sex ratio (male to female)**	8.53	10.44	0.91^1^
Average years of education	9.88 (3.71)	3.45 (4.42)	5.78^2^
Fraction of sample identifying as literate (able to read and write)	0.89	0.45	0.615^1^

Notes: Standard deviations in parentheses. Adapted from Bell et al. (2016)

* Median age

** Statistics on sex ratio from this study and the nationally and divisionally representative data from Ahmed (2013) are not directly comparable with official estimates of sex ratio (e.g., those from CIA 2016) since the latter represent estimates of the ratio of males to females in the population, while the former represent either ratios of male to female household heads (Ahmed 2013) or male to female study participants (this study).^1^ (CIA 2016).^2^ (UIS.Stat 2018)

### Experimental design

We constructed a set of 46 different short survey tasks, largely inspired by the 2011 Bangladesh Integrated Household Survey (BIHS; Ahmed 2013) with tasks common to such surveys—crop production, farm labor allocation, the experience of shocks, income, consumption, and the experience of illnesses, among others—as well as several tasks that engaged the unique capabilities of the smartphone, such as the mapping and reporting on tube well and latrine facilities available in the respondents’ geographic proximities. A small number of tasks describing variables that were not expected to change throughout the course of the year were completed only once, at the beginning of the experiment (e.g., household and farm structure). For the remaining tasks which were designed to collect information on variables expected to vary along the year, we prepared several variants, varying the frequency with which the task was given (weekly, monthly, or seasonally) as well as whether the data collection was also “crowdsourced,” in which the respondent was also requested to take on the role of a “citizen enumerator,” asking the survey question(s) of an additional individual (a family member, friend, neighbor, or even a stranger). In most cases, the length of the recall period matched the frequency of the task (e.g., weekly requests to report consumption over the past week; monthly requests to report over the entire month) but in some cases—such as food consumption—we elected to keep the same 1-week recall period irrespective of frequency. In other words, regardless of whether individuals received the food consumption survey task on a weekly basis or a monthly basis or a seasonal basis, the recall period was the same: 7 days. Each task was assigned a value from 1 to 5 “points,” with each point corresponding to a reward of 10MB of data and 5 Taka (~ 0.06USD). The value of each task was assigned keeping in mind three criteria: (a) the expected complexity of the task (a proxy for the expected cognitive burden that a particular task would impose on the participant, with more complex task being awarded more points); (b) the frequency with which the task was administered; and (c) the expected value of the data to the research team (with more valuable questions being worth more points). As illustrative examples of task value, the task of reporting whether any income had been saved in the last recall period had a value of 1, the task of reporting whether any agricultural goods had been sold, and to whom had a value of 3, while the task of identifying and photographing tube wells in one’s neighborhood had a value of 5. Participants could earn outright ownership of the handset they had been issued by accumulating at least 400 points throughout the duration of the experiment. A complete list of survey tasks and their value, along with the frequency, recall, and crowdsourcing variants prepared for each, is published in Bell et al. (2016). On average, participants received about 5-10 tasks each week, and our team paid an average of about USD65 per participant in data payments over the course of the 50-week experiment; including the USD45 Android device, the total cost per participant over the year was approximately USD110.

**Fig. 1:**
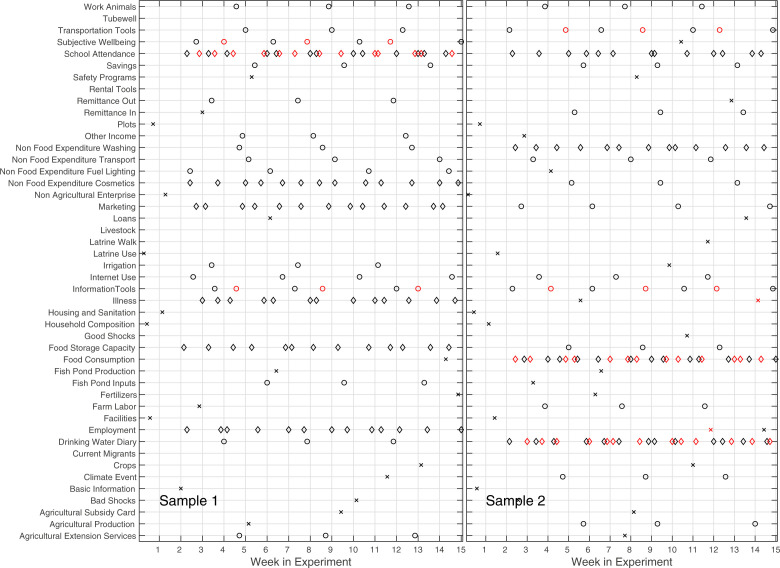
Two of the 20 unique phone setups, showing task schedule on a daily basis through the first 15 weeks of the experiment. One-time tasks are shown with an x, seasonal tasks with a circle, monthly tasks with a diamond, and weekly tasks with a triangle. Markers for tasks for the respondent are in black, while markers for crowdsourcing tasks are in red and bold-face

We designed 20 unique task schedules, each repeated on 24 handsets. Each schedule included exactly one version of each task (i.e., weekly, monthly, or seasonally; self only or crowdsourced). Across the different task schedules, parity in earnings potential was achieved by first randomly assigning task versions to the different schedules (e.g., a schedule might have been randomly assigned the weekly version of task *i*, but the monthly version of task *j*), and then making randomly selected pairwise switches of task versions between different schedules until the Gini coefficient (Jost 2006) for the potential earnings across schedules fell to below 0.001. All schedules include some weekly, some monthly, and some seasonal tasks, but each phone includes only one version of a particular task *i*; this design allows a standardized level of incentive, between-subjects comparisons of specific tasks, and within-subjects comparisons of engagement in weekly, monthly, and seasonal tasks in general. Between-subjects comparisons of specific tasks with different frequency draw together respondents using different phone setups (e.g., several different phone setups will include the weekly version of the food diary task), we have verified for the farm labor and food diary tasks (the central tasks included in the current analysis) that no significant differences in age, gender, education, literacy, or marital status exist among groups receiving different versions of these tasks (by one-way ANOVA at 95%). On average, the schedules solicited 5-10 tasks per week with a total potential value of around 30 points if all tasks were completed (two sample phone setups shown in Fig. 1).

**Fig. 2:**
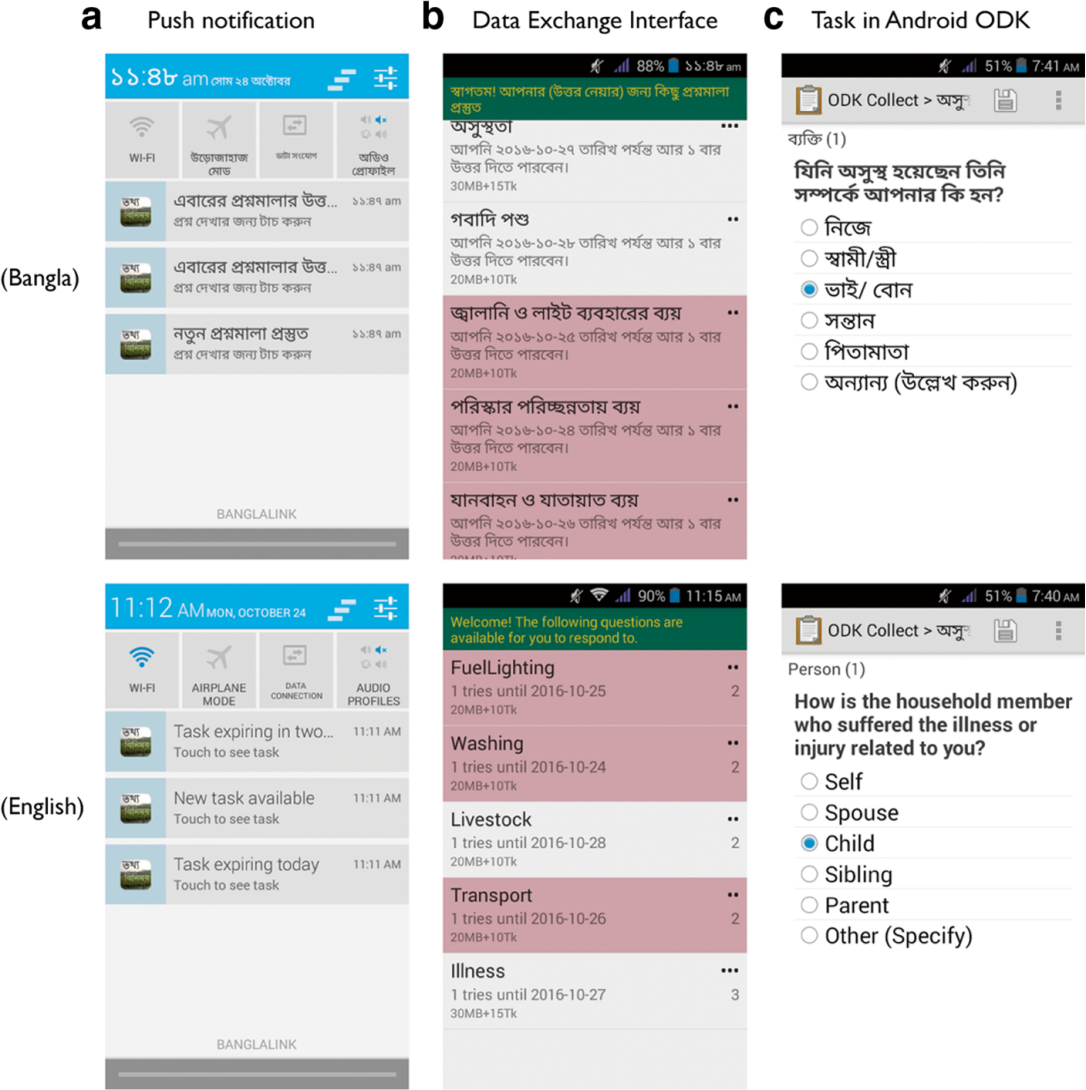
App experience for participants, reprinted from Bell et al. (2016). Participants receive a push notification that tasks are available (**a**). Tapping the notification or the app brings them to the Data Exchange interface (**b**), from which individual tasks are launched in Android ODK (**c**)

Survey tasks were implemented in ODK, whose interface is native to mobile devices and is designed around taps and swipes, and which provides easy switching across different languages, including Bangla. Though tasks implemented in ODK are intuitive to step through, the platform itself requires some specialized knowledge, so we also designed a custom interface called “Data Exchange” to streamline the process of notifying the participant that a task was available or was soon to expire, showing the task value, and launching the task itself (Fig. 2). All participants attended an intensive 1-day training session in which they (1) were introduced to the handset and learned about basic handset care; (2) learned some of the basic functions of the handset, such as how to make phone calls, send SMS text messages, access the calendar, use the camera, browse the internet; (3) were introduced to the Data Exchange application, how to access survey forms, etc.; (4) walked through specific examples of survey forms and the different types of data entry that might be expected (i.e., open-ended responses, selecting one or multiple items from a list, dates, etc.); (5) were introduced to the different survey form versions, again consisting of different combinations of frequency and self-versus self-plus crowdsourced responses; and (6) learned some of the “advanced” features of the handset, such as email, Facebook, YouTube.

### Analytical approach

In the present study, we examine the relative differences in within-subjects means and coefficients of variation (CVs) for different recall tasks, compared across weekly, monthly, and seasonal frequency variants. These two metrics were chosen to highlight two different dimensions of data and possibly allow us to distinguish the processes of (i) participants forgetting information on the one hand, and (ii) sampling methods missing information on the other. Specifically, we would expect the process of recall decay—systematically forgetting how many or how much—to manifest as a change in within-subjects mean, while we would expect the impact of missed events or intra-Period variation to manifest as a shift in within-subjects CV (with or without a concomitant change in within-subjects mean).

We include in our analysis all variables across our study where participants were asked to estimate a quantity (e.g., number of events experienced, amounts received or spent) over some recall period. These include a large number of agricultural labor estimations, as participants were asked to estimate labor use separately by task (planting, weeding, etc.), gender, and whether the laborer was a family member or a hired worker. Additionally, these include estimations of expenditures on different classes of non-food consumable items (transportation, fuel, sanitation, cosmetics), days of school missed for illness or other reasons, and food consumption.

Our analysis for each variable begins by first filtering for outliers. We separate responses into groups based on task frequency (since, e.g., responses summing labor over a week will be distributed differently than responses summing over a month), and then iteratively exclude responses lying more than 3 standard deviations from the mean, until no further outliers remain. We then restrict our analysis to respondents whose pattern of response is similar throughout the duration of the pilot by excluding respondents whose response count in weeks 3-26 (i.e., the first half of the experiment) differs from that in weeks 27-50 at 95% confidence interval by Kolmogorov-Smirnov test (i.e., their degree of participation does not wane over time, nor do they drop out of the pilot altogether). For this subset of participants, we then evaluate the within-subjects mean as the sum of the variable being recalled, divided by the total number of periods with a response (i.e., if the respondent answered 14 times along the experiment, then the mean is the sum divided by 14). The coefficient of variation is estimated similarly as the within-subjects standard deviation over the *n* periods with responses, divided by the within-subjects mean.

After calculating the group-level mean values for the within-subjects (over time) means and CVs for each variable, we normalize these group-level means by the largest observed group-level mean (across week, month, and season groups). Thus, for each variable, for each outcome of interest (mean and CV), we have an observation for each of the week, month, and season groups, the largest of which will be 1 (an individual’s observation normalized by itself). We identify statistical differences in the within-subjects means and COVs between groups using Kolmogorov-Smirnov tests at 95% confidence.

## Results

**Fig. 3:**
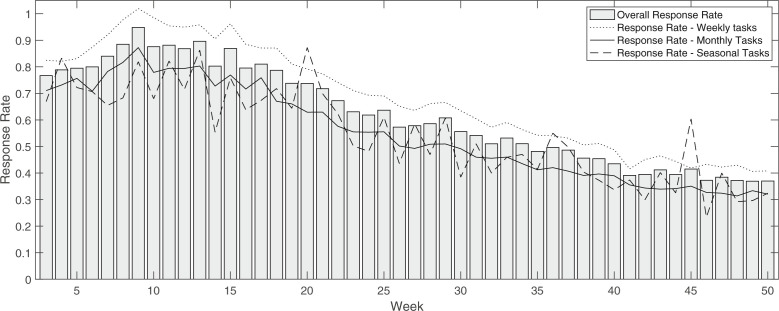
Average response rate to survey tasks along the experiment, both overall and broken apart by task frequency. Response rates to weekly tasks are significantly higher than monthly and seasonal response rates at 95% by Kolmogorov-Smirnov test

Our pilot spanned 50 weeks, with collection of basic information from participants (about household and farm structure, etc.) in the initial two weeks, and repeated tasks in the following 48 weeks. All data, tasks, and protocols are publicly available at doi:10.7910/DVN/HBQQVE (IFPRI and NYU 2018). As previously mentioned, incentives for participants’ continued engagement in the pilot included pre-paid mobile airtime and data top-ups, as well as credit toward ownership of the device, which many participants achieved within the first 4 months of the pilot. We observe significant drops in response rate along the experiment, with participation stabilizing in the final months of the pilot at a little under half of the peak response rates of about 85% (Fig. 3). A clear takeaway from this pattern is that the path to device ownership may be a powerful calibration parameter in future endeavors to better manage attrition. Importantly, we observe response rates to be significantly higher for weekly tasks than for either seasonal or monthly tasks along the entire experiment; this suggests higher net engagement with increased task frequency, rather than higher net burden to the participant as might naïvely be expected. We find no significant association of response rate with task value, by linear regression at 95% confidence. Importantly, we observe no significant association (by Kolmogorov-Smirnov test at 95% confidence) of age, gender, or education with attrition, defined as demonstrating a drop in engagement in the second half of the experiment in any one task (i.e., the exclusion criteria for analysis defined above in our methods section), with 175 households meeting this criteria.

**Fig. 4:**
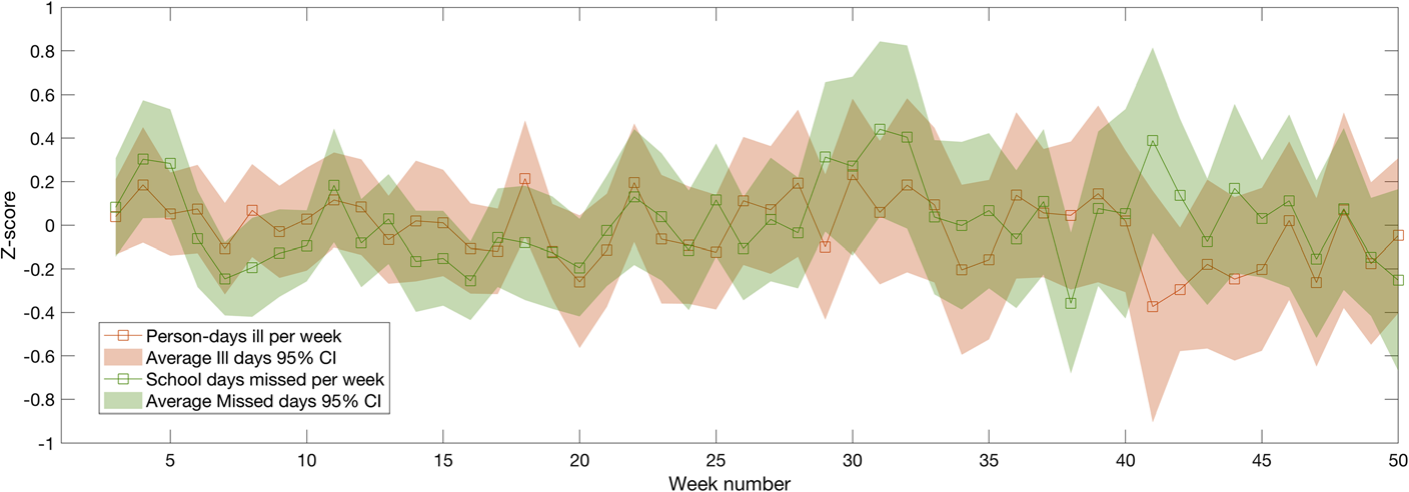
Weekly variation in experienced illness and school days missed, expressed as anomalies (*z*-scores)

Throughout the duration of the pilot, we observed a great deal of intra-annual variation in variables that commonly receive only point estimates in conventional surveys, such as illnesses and school absences (e.g., Fig. 4). In the following analyses, we quantify the differences in estimates of variables such as household labor and consumption that accrue from measuring only once per season, once per month, and once per week. Full descriptive statistics for these variables, broken apart by sampling frequency, are given as Table 2.

**Table 2:** Descriptive statistics for analyzed variables

Variable	Unit	N*	Weekly responses			Monthly responses			Seasonal responses		
			Mean*	SE*	n*	Mean*	SE*	n*	Mean*	SE*	n*
Unfiltered data											
Fuel spending	Taka/week	6800	627.80	(261.04)	154	264.07	(568.26)	184	49.32	(321.35)	141
Cosmetics spending		7264	264.41	(78.85)	177	64.70	(72.61)	205	19.36	(79.12)	103
Transport spending		2372	568.28	(170.99)	326	448.59	(1237.58)	159	67.80	(321.75)	39
Washing spending		2961	147.50	(38.39)	266	44.29	(49.02)	220	7.49	(22.88)	47
Crops sold		2648	515.86	(426.74)	142	188.06	(337.85)	135	90.64	(870.23)	95
Male family labor - plant	Person-hours/week	452	66.37	(36.65)	89	2.90	(5.75)	47	1.95	(6.93)	79
Male hired labor - plant		429	155.27	(112.76)	89	131.95	131.95	45	9.20	(28.54)	77
Female family labor - plant		236	31.73	(10.00)	59	4.94	(5.51)	27	1.27	(2.71)	60
Female hired labor - plant		224	299.83	(147.74)	56	52.93	(64.95)	24	57.20	(30.04)	54
Sick days	Days/week	3614	3.69	(1.42)	217	2.13	(4.95)	52	0.76	(7.11)	129
Missed school		3384	1.90	(0.58)	174	0.92	(1.16)	158	0.27	(1.79)	82
Cereals	kg/week	2917	4.59	(2.18)	168	4.61	(3.20)	50	3.00	(0.61)	54
Pulses		1362	4.32	(0.65)	161	0.44	(0.21)	38	1.24	(0.05)	51
Oils		1082	3.81	(3.17)	107	12.32	(2.54)	27	0.31	(0.05)	32
Vegetables		4855	2.98	(1.88)	195	5.68	(3.86)	66	6.50	(2.56)	78
Leafy vegetables		1356	2.46	(1.81)	170	0.76	(0.12)	40	2.51	(0.16)	51
Meat and egg		1777	2.54	(1.95)	185	12.66	(0.96)	51	1.17	(0.23)	64
Dairy		288	6.85	(4.56)	61	1.87	(0.08)	16	0.88	(0.03)	12
Big fish		903	2.16	(1.14)	157	1.05	(0.22)	35	22.38	(0.05)	28
Small fish		779	1.79	(1.08)	159	14.91	(0.13)	35	1.43	(0.04)	30
Spice		573	0.51	(0.26)	34	0.23	(0.00)	2	0.08	(0.00)	5
Drinks		139	1.26	(0.82)	42	0.45	(0.06)	8	0.79	(0.02)	9
Food outside of home		211	0.91	(0.37)	28	1.02	(0.09)	9	0.86	(0.00)	5
Fruits		615	2.82	(2.16)	100	1.43	(0.30)	21	2.34	(0.16)	32
Data screened for outliers and reduced/lost participation Fuel spending	Taka/week	6198	215.61	(50.99)	109	90.22	(91.77)	179	18.21	(60.94)	122
Cosmetics spending		6835	165.99	(27.22)	129	53.27	(50.73)	202	15.81	(45.73)	99
Transport spending		1147	279.86	(55.27)	221	84.26	(87.64)	150	39.02	(116.46)	37
Washing spending		1776	78.25	(13.22)	178	31.47	(27.88)	209	5.50	(14.90)	42
Crops sold		2128	10.43	(6.60)	122	3.14	(6.69)	122	2.01	(10.48)	84
Male family labor -plant	Person-hours/week	412	5.66	(2.71)	83	2.90	(5.75)	47	1.13	(4.09)	72
Male hired labor -plant		370	6.51	(3.13)	83	2.86	(6.14)	44	1.18	(3.10)	68
Female family labor -plant		208	3.32	(1.87)	52	1.68	(2.99)	24	0.95	(1.76)	59
Female hired labor -plant		171	5.94	(3.65)	42	3.85	(8.67)	19	2.41	(6.56)	46
Sick days	Days/week	3468	2.36	(0.88)	187	0.93	(1.27)	50	0.28	(1.41)	116
Missed school		3316	1.66	(0.54)	149	0.71	(1.01)	150	0.20	(0.93)	78
Cereals	kg/week	2863	1.69	(0.33)	124	1.40	(0.21)	47	2.24	(0.14)	52
Pulses		1298	0.34	(0.07)	150	0.27	(0.04)	37	0.20	(0.03)	42
Oils		993	0.21	(0.04)	90	0.24	(0.03)	21	0.29	(0.03)	31
Vegetables		4716	1.17	(0.31)	174	1.20	(0.29)	65	1.72	(0.25)	73
Leafy vegetables		1325	0.76	(0.15)	164	0.76	(0.12)	40	1.11	(0.17)	48
Meat and egg		1709	0.73	(0.19)	177	0.85	(0.16)	50	0.87	(0.08)	62
Dairy		280	0.67	(0.13)	60	0.75	(0.09)	14	0.88	(0.03)	12
Big fish		874	0.75	(0.16)	151	0.89	(0.23)	34	0.99	(0.05)	27
Small fish		755	0.65	(0.13)	156	0.52	(0.06)	33	0.61	(0.04)	29
Spice		544	0.16	(0.03)	32	0.23	(0.00)	2	0.08	(0.00)	5
Drinks		130	0.35	(0.09)	37	0.45	(0.06)	8	0.79	(0.02)	9
Food outside of home		195	0.38	(0.18)	24	0.52	(0.10)	8	0.86	(0.00)	5
Fruits		586	0.79	(0.24)	95	1.31	(0.18)	21	0.86	(0.17)	30

Key: *N*, total observations; *Mean*, average of within-subjects means; *SE*, average of within-subjects standard errors; *n*, number of subjects

### First analysis—comparing normalized mean and CV of summed labor, consumption, and income variables

**Fig. 5:**
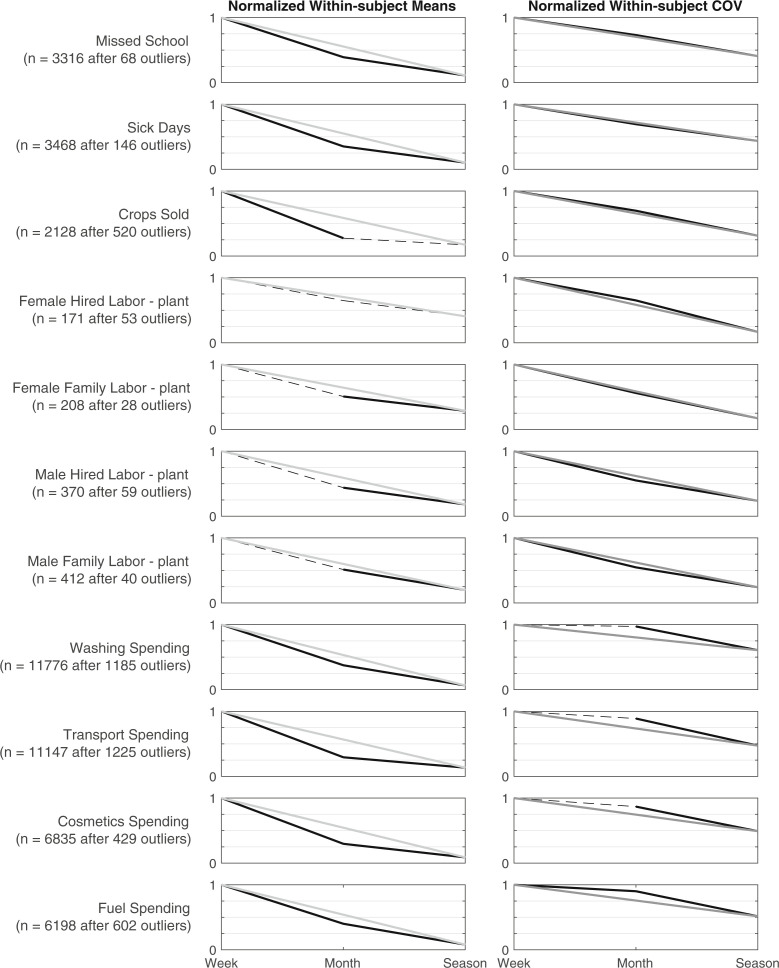
Normalized group-level averages of within-subjects average and coefficient of variation, across tasks asked weekly, monthly, and seasonally, with recall period == frequency (i.e., asked once a month to recall over past month). Additional labor variables included as Supplementary Material. Significant differences linked with solid lines, and non-differences linked with dashed lines; linkage from week to season shown in gray for clarity.

We compared the changes in within-subjects (i.e., along time) mean and coefficient of variation (CV) for all recall tasks involving counting or summing, across different recall periods of week, month, and season. In the case of the within-subjects mean, a negative relationship with recall length suggests a potential loss of information, perhaps due to salience. For within-subjects CVs, a negative relationship with recall length suggests missing intra-period variation (in other words, respondents tend to cognitively smooth out remembered experiences over longer periods of time). For most tasks that estimate spending, illness, or school absences, we observe significant decline in within-subjects mean as the recall period increases from week to month and to season (Fig. 5).We also observe significant declines in the within-subjects mean as the recall period increases from month to season for most labor estimations, though there are few differences as the recall period increases from week to month. We observe significant declines in within-subjects CVs for most labor tasks, as well as illnesses and school absences as the recall period increases from week to month to season, while observing a declining within-subjects CV for expenditures only as the recall period increases from month to season.

Taken together, these findings suggest that while there may be week-to-week variation in labor use, it is generally well recalled over the preceding month. In contrast, while spending may be similar from week to week, it is much more difficult to recall spending over the last month. To add even further contrast, illnesses, and school absences may vary from week to week and also be difficult to recall over a month recall period.

### Second analysis—comparing normalized mean and CV of summed food consumption variables

**Fig. 6:**
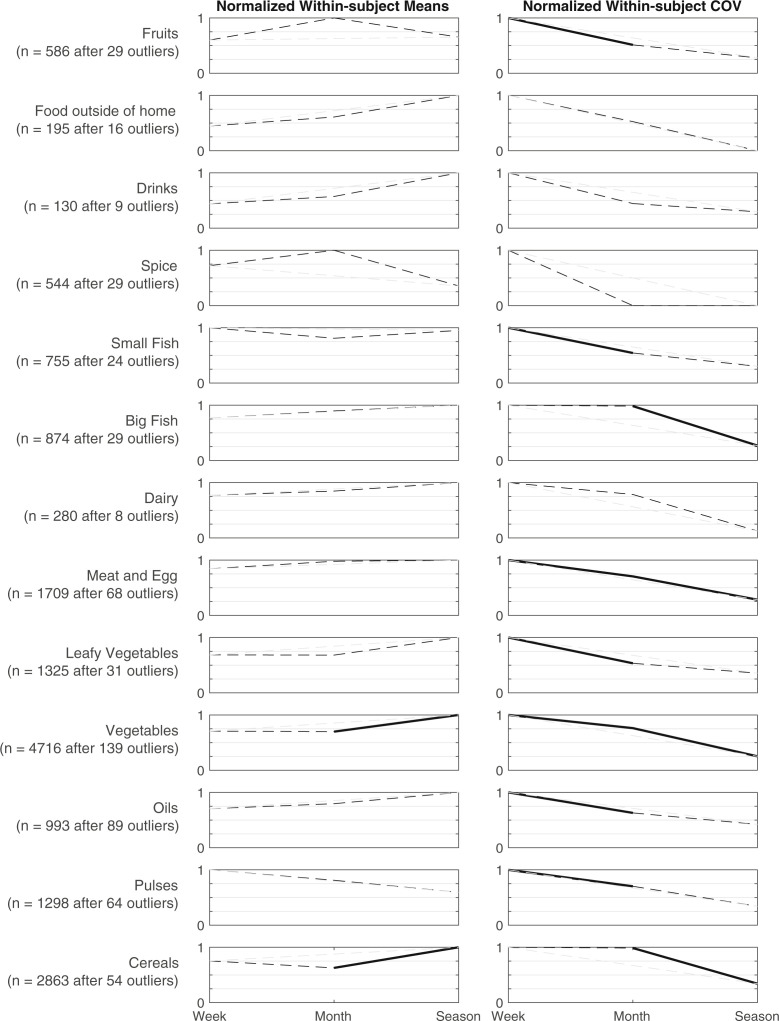
Normalized group-level averages of within-subjects average and coefficient of variation, across tasks asked weekly, monthly, and seasonally, with recall period == 1 day for all tasks (i.e., asked once a month to recall over past day only). Significant differences linked with solid lines, and non-differences linked with dashed lines; linkage from week to season shown in gray for clarity

Our pilot also included a food consumption diary, but in this case, the recall period was set to the preceding 1-day (24 h) period irrespective of the frequency with which this task was administered. The rationale behind this decision was that food consumption would be difficult to recall with any real degree of precision over a period longer than one week. In this case we would not necessarily expect significant differences in within-subjects mean across sampling frequency. In general, we do not observe any differences in the within-subjects means over response frequency, with two exceptions. Specifically, we find differences in the within-subjects means on consumption of both cereals and vegetables as the response frequency decreased from once per month to once per season (Fig. 6). It might be tempting to ascribe this as simply a weak signal of missed intra-period variation manifesting as a change in mean, but that would be better captured by the within-subjects CV, rather than the within-subjects mean. When we consider the CV, we do in fact observe a number of significant declines in CV in the food consumption diary with less frequent responses, with declining within-subjects CV as the response frequency drops from weekly to monthly for pulses, oils, vegetables and greens, fruits, meat and eggs, and small fish. We also observe declining within-subjects CVs for consumption of cereals, vegetables, meat and eggs, and big fish as the response frequency declines from monthly to seasonal. Thus, for many food consumption items, it seems apparent that less frequent data collection misses a great deal of variation that occurs in the intervening period, despite the fact that food consumption over the past day may not look that different on average, regardless of when a respondent is asked.

## Discussion and conclusions

Our design in this study—randomizing the frequency with which a respondent was given a particular task—was meant to inform the important question of whether there is value to asking certain questions more often, and if so, to identify which questions should be asked with a higher frequency. Our focus was primarily on quantitative data, which are arguably more prone to recall biases given the presumption of their specificity and objectivity, as well as humans’ difficulty in remembering such specific details with precision. Across our set of tasks, survey items requiring quantity responses fell into a relatively small number of categories, namely on-farm labor, employment and income, household experiences (such as illnesses and school absences), and consumption (food and non-food expenditures). Consequently, our analysis focused on identifying differences in either mean or relative variation in responses among participants depending upon the length of time over which they were asked to recall, or, in the case of food consumption, the frequency with which they were asked to provide 1-week recall of household food consumption. Our sample was purposively constructed to capture likely early adopters of smartphone technologies in this part of rural northern Bangladesh and skewed toward younger, more educated, and more male participants than a representative sample. Arthi et al. (2018) recently found recall bias to be lower among the more educated in a high-frequency telephone survey experiment in Tanzania. To the extent that such results are generalizable to rural contexts like our Rangpur sample, this suggests that differences in recall identified in our study might be even further pronounced (and the method of smartphone-based data collection even more worthwhile to develop) for the broader population.

Among our findings are several key messages about how these categories of tasks might best be asked. We observed significant declines in recalled average labor use as the response frequency increased from seasonally to monthly, but did not generally see changes in recalled average as frequency rose from monthly to weekly. We did observe significant differences in variation in the shift from monthly to weekly. Taken together, these findings suggest that although week-to-week use of farm labor may differ considerably, it remains prominent enough in respondents’ minds (due perhaps to expense, or predictability from year to year) that their ability to recall it does not decay within a relatively short period, such as a month. We infer from this finding that survey tasks focused on labor use could be administered once monthly without much loss in data precision, but that surveys only administered once a season (or, more likely, even less frequently) may significantly underestimate labor use.

Over all response frequencies, we observed significant changes in recalled average for all non-food expenditure tasks (i.e., spending on fuel, cosmetics, and household needs), as well as for those capturing the experiences of illness and school absences. In the case of expenditures, we did not observe significant differences in CV between weekly and monthly recall, though we did in the case of illnesses and school absences. These results suggest that consumption expenditures may be relatively consistent from week to week, but actual expenditure items may not be salient enough to be precisely recalled even after only 1 month. Even though the experience of illness and sick days was highly variable from week to week, it too was not salient enough to be well recalled by the end of a month. In both of these cases, we infer that measurement at a weekly scale would provide significantly different measurements than at any lower frequency.

We also included tasks in our pilot that standardized the recall period but still randomized the frequency at which they were asked, as in our food diary task (with a recall period of 24 h). Here, we did not expect to see significant differences in recalled average, and generally did not. We did, however, observe significant week-to-week variation in food consumption that would otherwise be missed by any assessment at lower frequency. This particular finding recasts the validity of the food diary (of the last day or week, e.g.) as a comparative measure of food security across any large sample (e.g., Ahmed et al. 2013), as it implies that the food consumption experience of those respondents near the beginning of a survey campaign could be utterly incomparable to that of the respondents visited toward the end, some weeks, or even months later. To some degree, problems such as this can be managed in very large studies by spreading data collection along a year or other period of interest and randomizing the order in which village clusters are visited (as recommended by the Inter-Agency and Expert Group on Food Security, Agricultural and Rural Statistics; IAEG-AG 2018); however, while incurring the kinds of logistic expense in trainings or fuel highlighted in our introduction, this approach also introduces spurious between-subjects variation that could limit the kinds of analysis possible. Our proposed approach, in which more frequent engagement also brought higher response rates, could be an alternative approach to this problem.

More broadly, we have demonstrated a feasible and cost-effective means of engaging respondents in a rural area to create a true socio-economic “baseline” (such as in Fig. 3), against which both inter-temporal as well as spatial anomalies can be identified. Further, we have applied a means of identifying the lowest appropriate frequency of data collection for specific tasks. Taken to scale, the regular observation of rural decisions and life experiences—including labor, consumption, the experience of illnesses or other shocks, to name a few—alongside the regular observation of the natural environment allows for research into coupled human-environment interactions and challenges that was previously not possible. Instead of only collecting detailed data as a “fire alarm response” (McCubbins and Schwartz 1984) to an experienced disaster such as a flood, drought, or cyclone, we can also examine the disasters that are never even experienced—contexts where existing infrastructure or institutions buffer against the anomalous shocks to natural systems, at which we would not otherwise have been looking. This is a critical advance, as it allows for proper identification of the roles that such institutions and infrastructure play in building or enhancing resilience to environmental variation and change.

In fairness, high-frequency data that capture temporal or spatially explicit variations in outcome measures may not be relevant for all research questions or policy evaluations. In many cases, one-shot “snapshot” surveys may suffice. However, for emerging research on social-environmental interactions, having social data at approximately the same regularity as natural environment data can shed a great deal of light onto previously unobserved—and in many cases unobservable—behaviors. The methods outlined in the present study draw together key building blocks—namely, an expanding global mobile broadband network, toolkits for data collection that are native to mobile devices, and a methodology for engaging rural participants—that we believe can be foundational in making regular, spatially explicit, mobile-based data collection the new normal in human-environment research in future. A critical next step for our research and for others engaging in unsupervised, mobile-based data collection is validation—studies that mirror mobile-based experimental designs with comparable telephone, traditional diary, or enumerated in-person surveys in order to identify similarities and contrasts, and to link smartphone-based findings with the history of conventional data collection.

## Footnotes

1 As an illustrative example of geographically representative data collection efforts, consider integrated household survey efforts (e.g., Malawi 2012; Ahmed 2013) or demographic censuses (e.g., NSO 2010; Zambia 2013)

2 As a very specific example, one of the authors was previously engaged in conducting a follow-up survey as part of a multi-year randomized controlled trial (RCT) in Bangladesh. Data collection was delayed for previously unforeseen reasons, so that by the time the enumeration team was in the field, data collection would span a period of time both before and during the 2014 Ramadan season, thereby invalidating any estimation of treatment effects on household food consumption.

3 We use the term “diary” to connote recalling events of a very recent nature, such as the past day (e.g., as in the standard use of “food consumption diaries”), unlike the stricter definition used in Beegle et al. (2012b) of event-driven logging of consumption or labor, as close to the moment of occurrence as possible.

4 Initial study recruitment was 480 participants, but with attrition, fewer than 480 participants remained engaged throughout the study—see “Results”.

## Notes

**Funding information** This work was supported by the Cereal Systems Initiative for South Asia (CSISA) of the Consultative Group on International Agricultural Research (CGIAR), with generous funding provided by the United States Agency for International Development (USAID), and the Bill and Melinda Gates Foundation.

**Publisher’s note** Springer Nature remains neutral with regard to jurisdictional claims in published maps and institutional affiliations.
