# Supplementary material for: Assessing recall bias and measurement error in high-frequency social data collection for human-environment research
Source: Popul Environ. 2019 Feb 7;40:325–45. doi: 10.1007/s11111-019-0314-1 (PMC7745111; doi:10.1007/s11111-019-0314-1)
Supplement: Supplementary file 1 [file PE-40-325-s001.docx]

**Supplemental Figures – Additional Labor Variables**


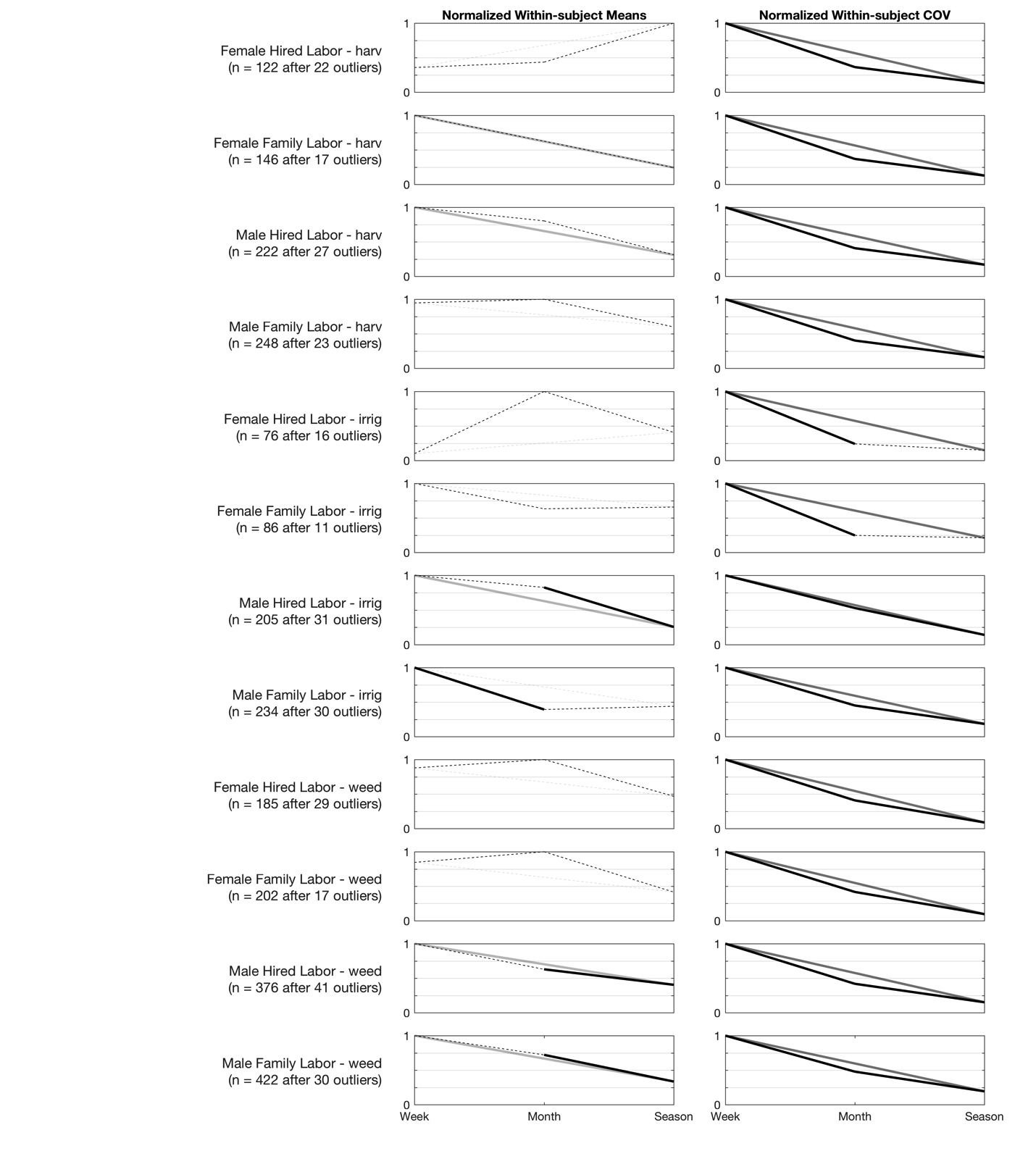


**Figure A1: Normalized group-level averages of within-subject average and coefficient of variation for additional labor tasks (harvesting, irrigation, and weeding), across tasks asked weekly, monthly, and seasonally, with recall period == frequency (i.e., asked once a month to recall over past month). Significant differences linked with solid lines, and non-differences linked with dashed lines; linkage from Week to Season shown in gray for clarity.**


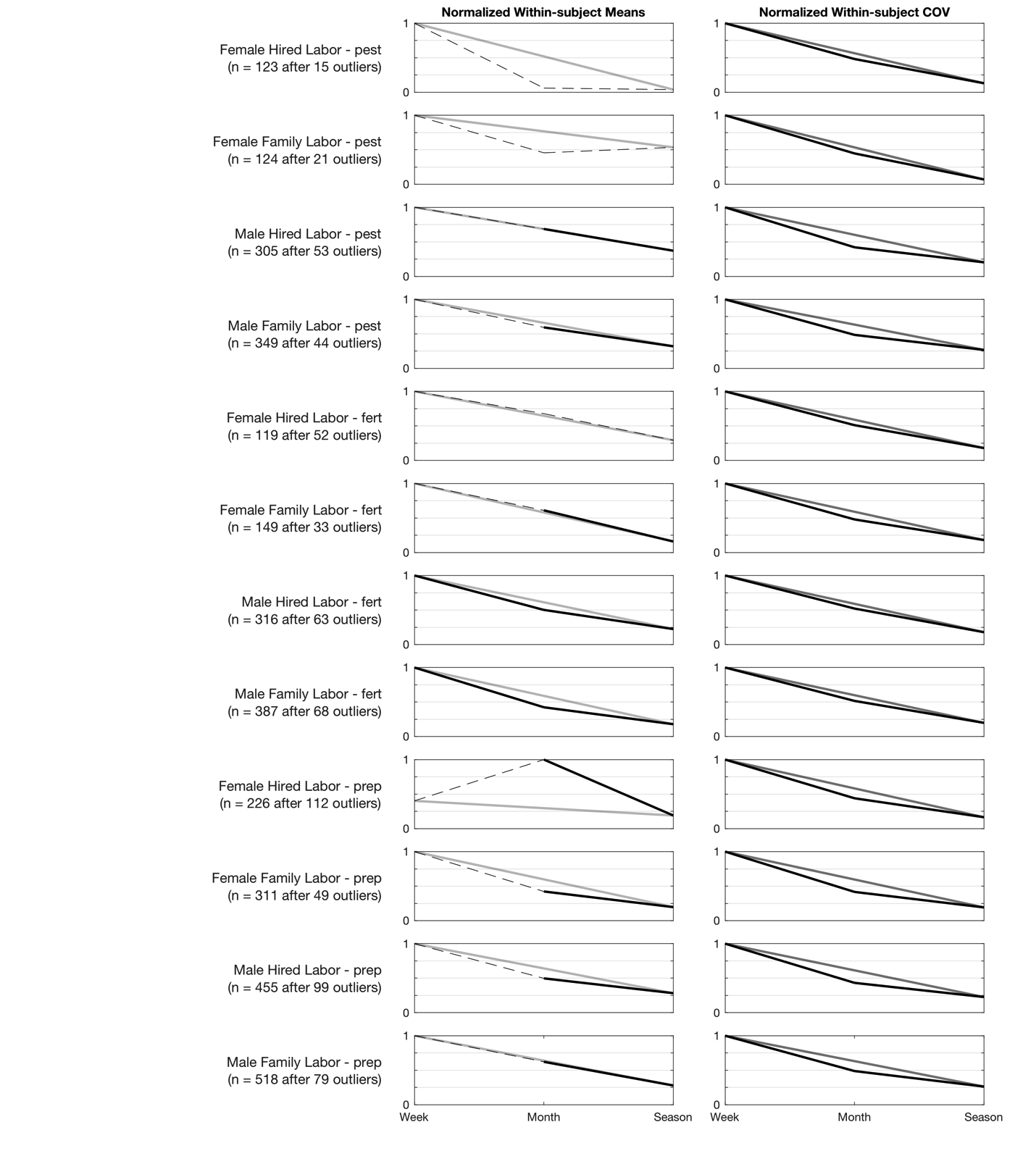


**Figure A2: Normalized group-level averages of within-subject average and coefficient of variation for additional labor tasks (pesticide application, fertilizer application, and land preparation), across tasks asked weekly, monthly, and seasonally, with recall period == frequency (i.e., asked once a month to recall over past month). Significant differences linked with solid lines, and non-differences linked with dashed lines; linkage from Week to Season shown in gray for clarity.**

Table A1: Descriptive statistics for analyzed variables (Part 1)

|  |  | **Unfiltered data** | | | | | | | | | |
| --- | --- | --- | --- | --- | --- | --- | --- | --- | --- | --- | --- |
|  |  |  | *Weekly responses* | | | *Monthly responses* | | | *Seasonal Responses* | | |
| Variable | Unit | N* | Mean* | SE* | n* | Mean* | SE* | n* | Mean* | SE* | n* |
| Male Family Labor - prep | Person-hours / week | **597** | 54.83 | (14.52) | 105 | 7.76 | (22.41) | 47 | 6.59 | (32.56) | 92 |
| Male Hired Labor - prep |  | **554** | 53.71 | (28.78) | 98 | 39.95 | (150.11) | 49 | 10.86 | (94.57) | 89 |
| Female Family Labor - prep |  | **360** | 22.52 | (7.30) | 73 | 9.70 | (26.98) | 34 | 2.95 | (25.42) | 68 |
| Female Hired Labor - prep |  | **338** | 514.84 | (95.41) | 68 | 79.08 | (193.03) | 33 | 28.38 | (122.15) | 63 |
| Male Family Labor - fert |  | **455** | 31.74 | (29.53) | 93 | 3.99 | (9.44) | 42 | 0.91 | (3.98) | 67 |
| Male Hired Labor - fert |  | **379** | 37.71 | (36.03) | 80 | 6.75 | (19.99) | 41 | 4.13 | (15.31) | 61 |
| Female Family Labor - fert |  | **182** | 45.07 | (42.15) | 47 | 3.89 | (5.02) | 25 | 1.60 | (14.10) | 34 |
| Female Hired Labor - fert |  | **171** | 404.32 | (396.31) | 43 | 388.05 | (1536.89) | 25 | 8.47 | (11.53) | 31 |
| Male Family Labor - pest |  | **393** | 18.26 | (16.36) | 73 | 6.63 | (14.74) | 38 | 0.98 | (5.97) | 48 |
| Male Hired Labor - pest |  | **358** | 31.76 | (28.34) | 62 | 34.22 | (59.32) | 35 | 2.41 | (28.00) | 47 |
| Female Family Labor - pest |  | **145** | 13.31 | (11.80) | 37 | 1.69 | (1.32) | 19 | 5.95 | (0.15) | 23 |
| Female Hired Labor - pest |  | **138** | 193.21 | (159.70) | 33 | 22.37 | (9.66) | 19 | 35.01 | (13.90) | 15 |
| Male Family Labor - weed |  | **452** | 14.24 | (12.12) | 94 | 5.39 | (11.14) | 41 | 3.40 | (12.40) | 55 |
| Male Hired Labor - weed |  | **417** | 41.43 | (13.58) | 95 | 44.69 | (166.75) | 39 | 16.83 | (67.87) | 55 |
| Female Family Labor - weed |  | **219** | 15.30 | (13.28) | 56 | 15.78 | (55.82) | 21 | 4.64 | (52.58) | 34 |
| Female Hired Labor - weed |  | **214** | 43.69 | (41.90) | 54 | 188.65 | (99.10) | 25 | 31.52 | (38.49) | 34 |
| Male Family Labor - irrig |  | **264** | 5.98 | (3.93) | 48 | 3.14 | (6.75) | 27 | 9.09 | (18.90) | 26 |
| Male Hired Labor - irrig |  | **236** | 29.60 | (27.08) | 43 | 32.39 | (85.33) | 25 | 712.12 | (140.82) | 26 |
| Female Family Labor - irrig |  | **97** | 26.33 | (24.09) | 17 | 2.79 | (5.08) | 15 | 3.09 | (16.97) | 13 |
| Female Hired Labor - irrig |  | **92** | 25.96 | (15.45) | 19 | 30.68 | (27.11) | 11 | 47.73 | (62.84) | 13 |
| Male Family Labor - harv |  | **271** | 36.66 | (30.81) | 78 | 6.63 | (9.08) | 22 | 73.28 | (7.51) | 34 |
| Male Hired Labor - harv |  | **249** | 29.85 | (12.11) | 75 | 5.61 | (9.05) | 23 | 25.37 | (30.56) | 34 |
| Female Family Labor - harv |  | **163** | 34.62 | (32.30) | 59 | 6.30 | (9.23) | 17 | 4.65 | (6.78) | 26 |
| Female Hired Labor - harv |  | **144** | 75.07 | (67.04) | 56 | 126.80 | (30.31) | 13 | 35.01 | (23.86) | 21 |
|  |  |  |  |  |  |  |  |  |  |  |  |
|  | **Key: N - Total observations; Mean - Average of within-subject means; SE - Average of within-subject standard errors; n - number of subjects** | | | | | | | | | | |

Table A1: Descriptive statistics for analyzed variables (Part 2)

|  |  | **Data screened for outliers and reduced/lost participation** | | | | | | | | | |
| --- | --- | --- | --- | --- | --- | --- | --- | --- | --- | --- | --- |
|  |  |  | *Weekly responses* | | | *Monthly responses* | | | *Seasonal Responses* | | |
| Variable | Unit | N* | Mean* | SE* | n* | Mean* | SE* | n* | Mean* | SE* | n* |
| Male Family Labor - prep | Person-hours / week | **518** | 5.58 | (3.04) | 97 | 3.47 | (5.71) | 45 | 1.56 | (7.46) | 81 |
| Male Hired Labor - prep |  | **455** | 5.02 | (2.45) | 87 | 2.50 | (4.06) | 45 | 1.42 | (6.02) | 79 |
| Female Family Labor - prep |  | **311** | 4.93 | (2.62) | 68 | 2.08 | (3.64) | 29 | 0.97 | (3.34) | 63 |
| Female Hired Labor - prep |  | **226** | 3.55 | (2.37) | 49 | 8.84 | (11.86) | 29 | 1.68 | (5.94) | 48 |
| Male Family Labor - fert |  | **387** | 2.87 | (1.66) | 86 | 1.22 | (2.57) | 39 | 0.51 | (1.97) | 61 |
| Male Hired Labor - fert |  | **316** | 2.52 | (1.40) | 70 | 1.26 | (2.64) | 40 | 0.56 | (1.61) | 51 |
| Female Family Labor - fert |  | **149** | 2.85 | (2.09) | 40 | 1.74 | (2.86) | 24 | 0.45 | (1.85) | 30 |
| Female Hired Labor - fert |  | **119** | 2.18 | (1.11) | 31 | 1.48 | (3.64) | 19 | 0.64 | (2.40) | 24 |
| Male Family Labor - pest |  | **349** | 2.47 | (1.14) | 68 | 1.47 | (2.03) | 34 | 0.79 | (2.74) | 48 |
| Male Hired Labor - pest |  | **305** | 2.46 | (1.24) | 58 | 1.69 | (2.42) | 28 | 0.91 | (2.32) | 45 |
| Female Family Labor - pest |  | **124** | 1.62 | (0.96) | 32 | 0.74 | (1.23) | 16 | 0.87 | (0.16) | 22 |
| Female Hired Labor - pest |  | **123** | 41.52 | (27.45) | 29 | 2.52 | (5.97) | 18 | 1.60 | (0.71) | 12 |
| Male Family Labor - weed |  | **422** | 4.27 | (2.43) | 92 | 3.08 | (5.93) | 40 | 1.43 | (6.23) | 48 |
| Male Hired Labor - weed |  | **376** | 5.17 | (2.46) | 93 | 3.25 | (5.16) | 39 | 2.09 | (5.58) | 49 |
| Female Family Labor - weed |  | **202** | 3.44 | (2.48) | 52 | 4.07 | (8.61) | 20 | 1.69 | (3.86) | 31 |
| Female Hired Labor - weed |  | **185** | 3.91 | (2.76) | 47 | 4.44 | (9.10) | 21 | 2.08 | (3.45) | 29 |
| Male Family Labor - irrig |  | **234** | 3.53 | (1.89) | 38 | 1.39 | (2.23) | 22 | 1.55 | (2.34) | 22 |
| Male Hired Labor - irrig |  | **205** | 3.67 | (2.07) | 37 | 3.03 | (5.99) | 23 | 0.94 | (1.42) | 19 |
| Female Family Labor - irrig |  | **86** | 3.60 | (2.16) | 14 | 2.27 | (2.59) | 14 | 2.37 | (1.71) | 12 |
| Female Hired Labor - irrig |  | **76** | 3.07 | (1.74) | 16 | 30.68 | (27.11) | 11 | 12.49 | (68.08) | 12 |
| Male Family Labor - harv |  | **248** | 5.67 | (3.34) | 74 | 5.99 | (6.75) | 22 | 3.59 | (7.98) | 32 |
| Male Hired Labor - harv |  | **222** | 6.22 | (3.58) | 69 | 5.00 | (6.83) | 23 | 1.94 | (8.85) | 27 |
| Female Family Labor - harv |  | **146** | 4.84 | (2.97) | 57 | 3.03 | (3.02) | 14 | 1.18 | (3.00) | 21 |
| Female Hired Labor - harv |  | **122** | 6.39 | (3.13) | 51 | 7.81 | (13.10) | 11 | 17.93 | (25.05) | 20 |
|  |  |  |  |  |  |  |  |  |  |  |  |
|  | **Key: N - Total observations; Mean - Average of within-subject means; SE - Average of within-subject standard errors; n - number of subjects** | | | | | | | | | | |
